# Deep-ocean mixing driven by small-scale internal tides

**DOI:** 10.1038/s41467-019-10149-5

**Published:** 2019-05-08

**Authors:** Clément Vic, Alberto C. Naveira Garabato, J. A. Mattias Green, Amy F. Waterhouse, Zhongxiang Zhao, Angélique Melet, Casimir de Lavergne, Maarten C. Buijsman, Gordon R. Stephenson

**Affiliations:** 1 Ocean and Earth Science, University of Southampton, National Oceanography Centre, Southampton, SO14 3ZH UK; 2 School of Ocean Sciences, Bangor University, Menai Bridge, Anglesey, LL57 2DG UK; 3 Scripps Institution of Oceanography, University of California, San Diego, La Jolla, CA 92037 USA; 4 Applied Physics Laboratory, University of Washington, Seattle, WA 98105 USA; 5 Mercator Ocean, Ramonville Saint-Agne, 31520 France; 6 LOCEAN Laboratory, Sorbonne Université-CNRS-IRD-MNHN, Paris, 75005 France; 7 University of Southern Mississippi, Stennis Space Center, Hattiesburg, MS 39556 USA; 8 Present Address: LOPS, Plouzané, Bretagne, France

**Keywords:** Physical oceanography, Physical oceanography

## Abstract

Turbulent mixing in the ocean is key to regulate the transport of heat, freshwater and biogeochemical tracers, with strong implications for Earth’s climate. In the deep ocean, tides supply much of the mechanical energy required to sustain mixing via the generation of internal waves, known as internal tides, whose fate—the relative importance of their local versus remote breaking into turbulence—remains uncertain. Here, we combine a semi-analytical model of internal tide generation with satellite and in situ measurements to show that from an energetic viewpoint, small-scale internal tides, hitherto overlooked, account for the bulk (>50%) of global internal tide generation, breaking and mixing. Furthermore, we unveil the pronounced geographical variations of their energy proportion, ignored by current parameterisations of mixing in climate-scale models. Based on these results, we propose a physically consistent, observationally supported approach to accurately represent the dissipation of small-scale internal tides and their induced mixing in climate-scale models.

## Abstract (web summary)

The geography of deep-ocean mixing driven by internal tides is poorly constrained in ocean models. Here the authors unveil the global variability of energetic small-scale internal tides, combining an analytical model with satellite and in situ observations, paving the way to future parameterisations.

## Introduction

The deep ocean communicates with the atmosphere through a network of currents termed the meridional overturning circulation. Chokepoints of communication include upwelling currents across density stratification, maintained by turbulent diapycnal mixing. Decades of observations have revealed that the processes driving mixing exhibit prominent spatio-temporal variability, and are often energised in the proximity of complex topography^1^. In turn, recent theoretical and modelling investigations have shown that the spatial distribution of mixing strongly impacts the ocean state, highlighting an imperative to develop realistic and physically consistent representations of key mixing processes^2–4^. The generation and breaking of internal (or baroclinic) tides is a primary driver of deep-ocean mixing^5–7^. Lunisolar tides supply ~1 TW of mechanical energy to global internal tide generation outside of continental shelves^6^, which is approximately half of the 2 ± 0.6 TW required to fuel global turbulent dissipation and overturning^8,9^. Lunisolar tides lose their energy on interacting with major features of the seafloor topography, such as mid-ocean ridges and seamounts, and thereby transfer much of their energy to internal tides. Although the geography of internal tide generation is relatively well understood (as it depends, to first order, on well-known barotropic tidal currents and properties of the seafloor topography^10^), the waves’ subsequent evolution and eventual fate are highly uncertain. As a result, parameterisations of tidally driven mixing in climate-scale ocean models are poorly constrained^7^, and mechanistic descriptions of the association between mixing and overturning suffer from fundamental knowledge gaps^3^.

Internal tides span a wide range of vertical and horizontal scales, and it is common practice to cast them into a discrete set of normal modes with distinct structures determined by the local stratification^11^. Observations show that low-mode (i.e., large-scale) internal tides are able to propagate over one thousand kilometres from their generation site, such that their breaking may contribute to far-field oceanic background mixing or remote mixing hot spots. In contrast, high-mode (i.e., small-scale) internal tides are prone to direct breaking, often triggered by wave-wave interactions^12^, and thus drive mixing in the near field of their generation site. The dichotomous fate of low-mode and high-mode waves calls for quantification of the modal partitioning of internal tide generation. Critically, this modal partitioning is directly linked to the parameter *q*, the fraction of locally dissipated tidal energy to local barotropic-to-baroclinic tidal energy conversion. This parameter is a cornerstone of parameterisations of tidal mixing^2,13^ used in state-of-the-art climate-scale ocean models, such as the Community Climate System Model version 4 (CCSM4^14^) and the Nucleus for European Modelling of the Ocean (NEMO^15^). In these models, *q* adopts a constant value of 1/3, although the potential for significant spatial variability in *q* is acknowledged by several studies^16–18^.

In this paper, we present an estimate of the modal partitioning of global internal tide generation with a resolution of up to 50 modes, and show that its predictions are consistent with available observations of tidal energy conversion, radiation and mixing. We demonstrate that the generation of very high (>10) modes accounts for a remarkably large fraction (27%) of all tidal energy conversion. Contrary to current views, reflected in parameterisations of tidal mixing, near-field mixing, associated with locally generated high-mode internal tides, dominates tidal mixing on a global scale (>50%) and exhibits a strong geographical variability: the parameter *q* is widely distributed across values from 0 to 1. These findings have important implications for the representation of deep-ocean mixing and overturning in climate-scale ocean models, which we discuss.

## Results

### Energy budgets and evidence of high-mode generation

**Fig. 1:**
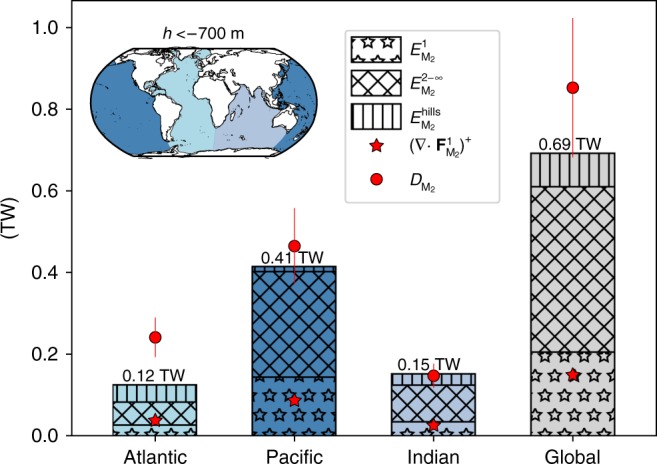
Regional and global energy budgets for the M_2_ tide. Stacked histograms represent energy conversion into mode 1 ($E_{{\mathrm{M}}_2}^1$, starred areas), modes 2-∞ ($E_{{\mathrm{M}}_2}^{2 - \infty }$, here excluding $E_{{\mathrm{M}}_2}^{{\mathrm{hills}}}$, diagonally hatched areas) and the contribution from abyssal hills ($E_{{\mathrm{M}}_2}^{{\mathrm{hills}}}$, vertically hatched areas). Note that $E_{{\mathrm{M}}_2}^{1 - \infty } = E_{{\mathrm{M}}_2}^1 + E_{{\mathrm{M}}_2}^{2 - \infty } + E_{{\mathrm{M}}_2}^{{\mathrm{hills}}}$. Red stars are the divergence of altimetry-derived M_2_ mode-1 energy flux, $(\nabla \cdot {\mathbf{F}}_{{\mathrm{M}}_2}^1)^ +$, and red bullets are the barotropic tide energy loss ($D_{{\mathrm{M}}_2}$). Error bars show ±20% of $D_{{\mathrm{M}}_2}$ as suggested in Egbert and Ray^26^ for deep-ocean integrals. The inset map shows the boundaries separating the different basins. Budgets are computed for seafloor depths greater than 700 m

We use a linear, semi-analytical model of barotropic-to-baroclinic tidal energy conversion based on the assumptions of subcritical topography and small tidal excursion^19–22^. The model takes into account the spectral shape of seafloor topography, the barotropic tidal currents and the frequencies of the system, and predicts the energy conversion as a function of wavenumber and azimuthal direction (see Methods). This spectral-space method relies on the same assumptions as real-space methods, which can also compute estimates of the modal partitioning of internal tide generation^23^. It however has the advantage of not predicting negative conversion rates^23,24^ that are difficult to interpret physically. The calculation is performed globally on a 1/2° grid and gives, at each grid point, the energy conversion $E_\omega ^n$ into mode *n* (with *n* ≥ 1) for a tide of frequency *ω*. The highest mode resolved by our model depends on the resolution of the bathymetric data set, latitude, and stratification at each location; at mid-latitudes, it is approximately 50. However, the global bathymetry product does not resolve abyssal hills (topographic features with lateral scales of *O*(1–10) km that populate mid-ocean ridges), yet these are responsible for non-negligible energy conversion^25^. In order to account for this, we complement our model with a published independent estimate of tidal energy conversion by abyssal hills^25^, hereafter denoted $E_{{\mathrm{M}}_2}^{{\mathrm{hills}}}$ (only computed for the semidiurnal tide M_2_). In the following, $E_{{\mathrm{M}}_2}^{{\mathrm{hills}}}$ is included in $E_{{\mathrm{M}}_2}^{n - \infty }$ for *n* ≥ 1, except in Fig. 1 and associated discussion. Our estimates of energy conversion are corrected wherever topographic slopes are supercritical, as done in preceding studies^23,25^ (see Supplementary Note 1). In the following, unless stated otherwise, we focus on the semidiurnal M_2_ tidal constituent, which accounts for the bulk of tidal energy conversion in the deep ocean^26^.

We assess the realism of our estimate of energy conversion with two independent observational data sets: the energy lost by the barotropic tide, $D_{{\mathrm{M}}_2}$, computed through an inverse analysis of satellite altimetric measurements; and the positive part of the mode-1 energy flux divergence, $(\nabla \cdot {\mathbf{F}}_{{\mathrm{M}}_2}^1)^ +$, also estimated from satellite altimetric data^27^ (see Methods). $(\nabla \cdot {\mathbf{F}}_{{\mathrm{M}}_2}^1)^ +$ quantifies the rate of generation of mode-1 internal tides. These altimetry-based data sets enable us to define regional and global budgets of the M_2_ tidal energy, and to validate the predictions of our semi-analytical model (Fig. 1). All terms are integrated for seafloor depths larger than 700 m, in order to exclude shallow topography where the supercritical-slope correction is important (see Supplementary Fig. 1).

**Fig. 2:**
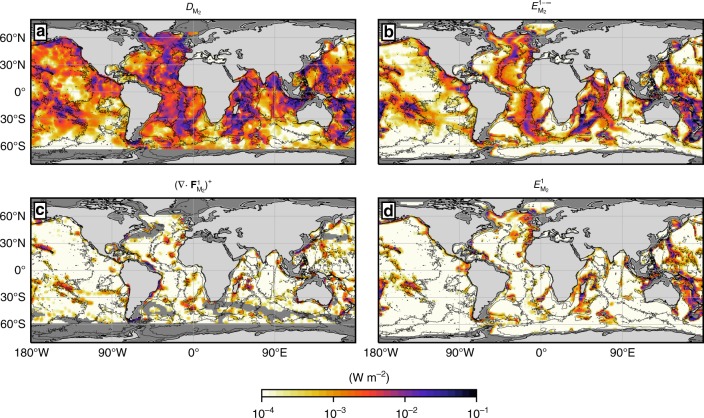
Geography of energy lost by the semidiurnal M_2_ barotropic tide and converted into internal tides. **a** Energy lost by the M_2_ barotropic tide, $D_{{\mathrm{M}}_2}$, computed from TPXO8^50^, and **b** barotropic-to-baroclinic tide energy conversion from the semi-analytical model, $E_{{\mathrm{M}}_2}^{1 - \infty }$. **c** Positive part of the divergence of M_2_ mode-1 horizontal energy flux $(\nabla \cdot {\mathbf{F}}_{{\mathrm{M}}_2}^1)^ +$ derived from satellite altimetry^27^ (areas where the mesoscale activity is too strong to recover the internal tide signal are masked in dark grey), and **d** barotropic-to-baroclinic tide energy conversion into mode 1 from the model, $E_{{\mathrm{M}}_2}^1$. Areas shallower than 700 m are shown in dark grey

The correspondence between observational estimates and our model’s predictions is notable and enables, for the first time, to accurately depict a budget for the energy lost by the barotropic tide, when decomposed into various basins and various contributing components. First, the bulk of the energy lost by the M_2_ barotropic tide is found to be converted into internal tides for all three major ocean basins: $E_{{\mathrm{M}}_2}^{1 - \infty }$ is within the error bars of $D_{{\mathrm{M}}_2}$ globally (692 GW vs. 853 ± 171 GW). The contribution of abyssal hills is crucial to close the budget of barotropic tide dissipation, as it represents 12% of the conversion, globally. Note that the marginally significant difference between $D_{{\mathrm{M}}_2}$ and $E_{{\mathrm{M}}_2}^{1 - \infty }$ in the Atlantic basin, suggestive of missing conversion, may be attributable to under-represented conversion by abyssal hills^25^. The geographical patterns of $D_{{\mathrm{M}}_2}$ and $E_{{\mathrm{M}}_2}^{1 - \infty }$ match throughout the global ocean (see Supplementary Note 2 and Supplementary Fig. 2), and reveal that M_2_ tidal energy conversion is amplified over mid-ocean ridges, seamounts, and continental shelf breaks (Fig. 2a, b).

Second, there is a close agreement between our model’s predictions ($E_{{\mathrm{M}}_2}^1$) and observational estimates ($(\nabla \cdot {\mathbf{F}}_{{\mathrm{M}}_2}^1)^ +$) of the rate of generation of M_2_ mode-1 internal tides, both globally and for each of the major ocean basins (Fig. 1). Mode 1 only accounts for 29% of M_2_ tidal energy conversion on a global scale. The relative importance of mode-1 is lower in the Atlantic and Indian basins, where mode-1 contributes 21 and 23% of all M_2_ tidal energy conversion, but is higher in the Pacific basin, where the fraction of mode-1 conversion is 35%. The enhanced generation of mode-1 internal tides in the Pacific Ocean stems from the basin’s comparative abundance of steep ridges and seamounts, which are conducive to the generation of low-mode baroclinic tides. Hot spots of mode-1 generation are co-located in $E_{{\mathrm{M}}_2}^1$ and $(\nabla \cdot {\mathbf{F}}_{{\mathrm{M}}_2}^1)^ +$ (Fig. 2c, d; Supplementary Note 3 and Supplementary Fig. 3): prominent sites include the Canary Islands, the Atlantis-Meteor Seamount complex, the Iberian shelf break and the Walvis Ridge in the Atlantic Ocean; the northern and southern edges of Madagascar in the Indian Ocean; and the Hawaiian and Polynesian Ridges, the Galápagos archipelago, the Tonga-Kermadec Ridge (north of New Zealand) and the Tasman Sea shelf break in the Pacific Ocean. Other likely sites of important M_2_ mode-1 internal tide generation south of 40 °S, such as the Kerguelen Plateau, could not be resolved in the observational data set due to contamination of the tidal signals by the region’s energetic mesoscale eddy field. All in all, both our model and the observations indicate that the bulk (71–82%) of M_2_ tidal energy conversion occurs in modes higher than 1. This result challenges the widespread view that mode 1 overwhelms tidal energy fluxes^28^.

**Fig. 3:**
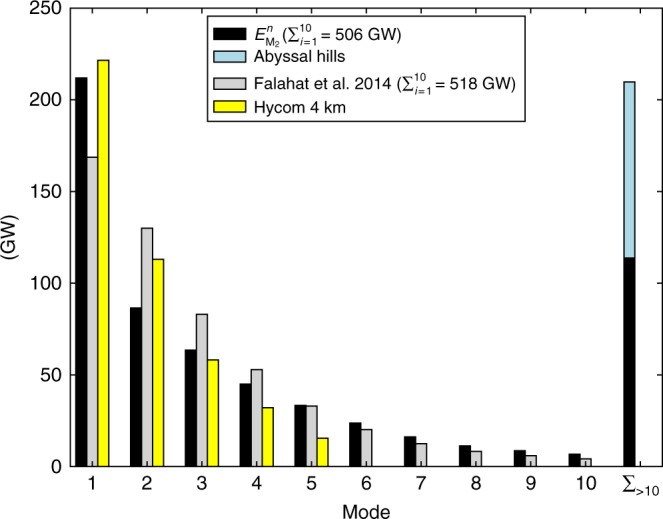
Histogram of energy conversion as function of mode number. Global energy conversion below 700 m into the first 10 modes, and for the sum of modes higher than 10 (rightmost bar), based on (black bars) our model complemented with (blue bar) the contribution of abyssal hills of Melet et al.^25^, (grey bars) Falahat et al.^23,60^, and (yellow bars) a global HYCOM simulation. Modes >10 were not computed in Falahat et al.^23^, and the HYCOM simulation does not resolve modes >5

The robustness of our model is further endorsed by two independent calculations of the modal partitioning of internal tide generation. Falahat et al.^23^ used a different approach to ours, but based on the same assumptions, to compute the M_2_ tidal energy conversion for the first 10 modes. Their modal distribution closely matches ours for this subset of modes (Fig. 3), with a global conversion into modes 1–10 (integrated up to 700 m) of 518 GW vs. 506 GW. Our model has slightly more energy in modes 6–10, which could be attributed to the better-resolved bathymetry dataset used (SRTM30-PLUS vs. ETOPO2) and the upgraded barotropic tide model used to derive barotropic tidal currents (TPXO8 vs. TPXO6.2). Our estimate for modes 1–5 also compares well to the internal tide generation diagnosed in a state-of-the-art global numerical simulation (Fig. 3) at a horizontal resolution of 4 km using the primitive-equation model HYCOM (see Methods). Modes higher than ~4 are only partially resolved in HYCOM as this model cannot adequately represent the horizontal and vertical internal wave lengths. While the consistency between our predictions and these independent estimates is reassuring, our results highlight that modes higher than 10, hitherto unresolved, account for a large fraction—27%, including the contribution of abyssal hills—of global M_2_ internal tide generation. The importance of modes higher than 10 is bolstered by the fact that the integrated conversion into modes 1–10 (506 GW) is too small to explain the observed energy lost by the M_2_ barotropic tide (853 GW, Fig. 1). Since bottom drag is only a minor player in the dissipation of the M_2_ barotropic tide in the deep ocean^29^, the missing energy sink must be attributed to the generation of mode >10 internal tides.

### Geographical variability of modal content

**Fig. 4:**
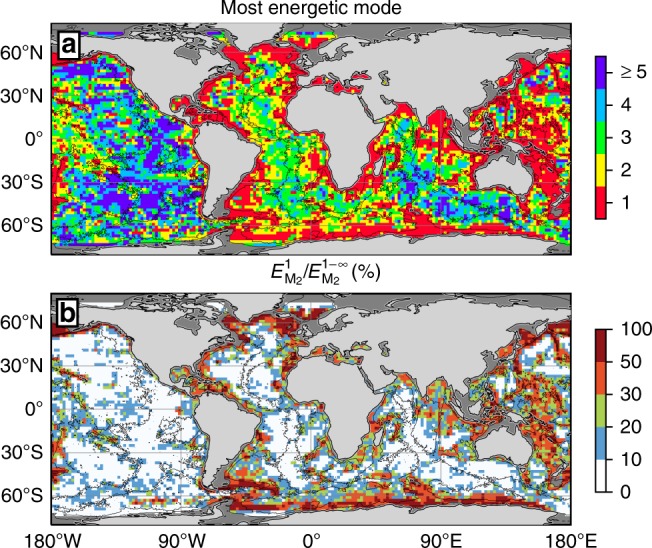
Geography of modal content of energy conversion. **a** Mode number *n* for which $E_{{\mathrm{M}}_2}^n$ is maximum, and **b** ratio of energy conversion into mode 1 to the total energy conversion for the M_2_ tide, shown as percentage. Variables are binned in 2 × 2° squares for visual purposes

The geographical variability of the modal partitioning of M_2_ tidal energy conversion is illustrated in Fig. 4. Continental shelf breaks, steep ridges and isolated seamounts stand out as preferential locations for mode-1 internal tide generation (Fig. 4a). In contrast, wide ridge systems, such as the Mid-Atlantic Ridge and the East Pacific Rise, systematically display a peak in the energy conversion continuum around modes 2–5. In the deep regions of the Pacific, the most energetic modes are often ≥5.

Although mode 1 is the most energetic mode on a global scale (Fig. 3), its fractional contribution to the total conversion, $E_{{\mathrm{M}}_2}^1/E_{{\mathrm{M}}_2}^{1 - \infty }$, exhibits pronounced geographical variability (Fig. 4b). Over continental shelf breaks and steep ridges, mode 1 can account for over 50% of the total M_2_ tidal energy conversion. For example, in the Hawaiian Ridge system, mode 1 contributes between 40 and 60% of the total conversion, in the range of the ratios estimated from observations^30^ and regional numerical simulations^31^. However, over most of the global ocean, mode 1 accounts for <30% of the M_2_ tidal energy conversion. Specifically, in wide regions of strong conversion such as the Mid-Atlantic Ridge and the East Pacific Rise, mode 1 contributes less than 10% of the total conversion. Considering that mode 1 may be the only mode capable of propagating far (>1000 km) from its generation site^22,32–34^, this modal partitioning of conversion suggests that most of the internal tide energy sourced in these regions dissipates within a short distance of generation sites.

### Implications for near-field dissipation

Areas where strong tidal energy conversion occurs span very different topographic structures, which affect the modal content of locally generated internal tides. Thus, strong geographical variability of near-field dissipation is expected, with high (small) rates where high (low) modes are preferentially generated. Here, we propose a new approach to assess near-field dissipation driven by the breaking of locally generated, high-mode internal tides, which can be used to construct parameterisations of near-field mixing in ocean models (see Discussion). Key to the approach is the definition of a critical mode *n* above which all modes are assumed to dissipate locally, i.e., within the grid point where conversion occurs. In turn, modes <*n* can propagate away and dissipate in the far field. The rate of near-field tidal dissipation is thus defined as *E*^*n*−∞^. Among the range of processes that may trigger a forward cascade and dissipation of internal tide energy, we consider only the dominant mechanism, i.e., wave-wave interactions^12^, which renders our estimate of near-field dissipation conservative. We take the attenuation time scale *τ*_*n*_ of mode *n* associated with wave-wave interactions to be proportional to 1/*n*^2^ (see Fig. 33 in Olbers^35^), which yields an attenuation length scale for that mode *L*_*n*_ = *c*_*n*_*τ*_*n*_ proportional to 1/*n*^3^, since the group velocity *c*_*n*_ is proportional to 1/*n*. Consequently, *L*_*n*_ ≈ *L*_1_/*n*^3^. Using numerical simulations and in situ data from a mooring array aligned with an internal tide beam emanating from the Hawaiian Ridge^36^, we estimate the characteristic attenuation length scale of mode 1, *L*_1_, to be between 700 and 1300 km. Although *L*_1_ is expected to vary geographically, depending notably on mesoscale activity and topographic scattering, we find that the critical mode number is weakly sensitive on *L*_1_ (see Supplementary Note 4 and Supplementary Figs. 4 and 5). On our global 1/2°-grid, mode 4 is found to be the critical mode. Although this method leads to a critical mode that is grid-size-dependent by construction, 1/2° is typical of climate-scale ocean models, and the critical mode can be adjusted to fit different grid sizes. Note that this estimate of near-field dissipation does not account for local breaking of low-mode internal waves occurring at steep ridges, e.g., through lee-wave radiation mechanisms^37,38^ or direct shear instability^39^, reinforcing the conservatism of our approach.

**Fig. 5:**
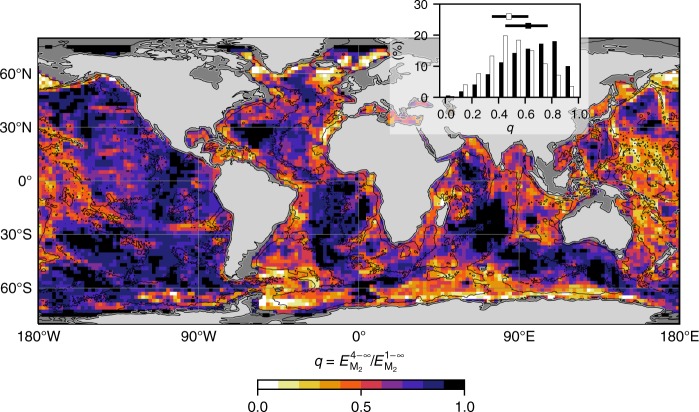
Geography of the ratio *q* of local energy dissipation to total energy conversion. The ratio *q* is here defined as $q = E_{{\mathrm{M}}_2}^{4 - \infty }/E_{{\mathrm{M}}_2}^{1 - \infty }$, and is binned in 2° × 2° squares for visual purposes. Inset bar-plot represents histograms of (black) seafloor area and (white) $E_{{\mathrm{M}}_2}^{1 - \infty }$ binned as a function of *q*, and error bars represent the 25th and 75th percentiles on both sides of the median (square)

The estimated rate of near-field dissipation may be used to quantify *q*, the fraction of locally dissipated energy to local tidal energy conversion. The definition *q* = *E*^4−∞^/*E*^1−∞^ is adopted here. The geographical structure of *q* reflects the spatial variability in the properties of seafloor topography (Fig. 5). Where high-mode internal tide generation is substantial, e.g., over the Mid-Atlantic Ridge and the East Pacific Rise, *q* is high and reaches values in the range 0.8–1.0. In such regions, the contribution of abyssal hills is critical to weight the conversion towards high modes and reach high *q* values. In contrast, where low-modes are preferentially generated, e.g., the Hawaiian Islands and other Pacific archipelagos, *q* displays smaller values of 0.3–0.5. The global-ocean probability density functions of seafloor area and M_2_ tidal energy conversion as a function of *q* (Fig. 5-inset) quantify the geographically variable significance of near-field dissipation. Although half of the global conversion occurs in regions where 0.35 < *q* < 0.62 (25th and 75th percentiles), half of the seafloor area features 0.45 < *q* < 0.77 (25th and 75th percentiles). Hence, viewed overall, our conservative, semi-analytical estimate of *q* highlights the strong spatial variability of near-field dissipation, and shows that, on average, *q* greatly exceeds the value of 1/3 generally assumed to date.

### In situ observational estimates of turbulent dissipation

Our model reveals that the generation of energetic high-mode internal tides is widespread across the global ocean. These high modes are characterised by a small group speed and a high vertical shear, which make them prone to breaking close to their generation site. We therefore expect a close relationship between the predicted near-field dissipation of internal tides and the in situ dissipation of turbulent kinetic energy. To evaluate this relationship, we compared our theoretical estimate of near-field dissipation for the eight principal tidal constituents, i.e., $E_{{\mathrm{all}}}^{4 - \infty }$ (see Methods), to in situ estimates of turbulent energy dissipation from a finescale parameterisation applied to hydrographic measurements^9^ and from microstructure observations^8^. Regions where the mean kinetic energy or the eddy kinetic energy are elevated (>200 cm^2^ s^−2^, visually chosen to discard western boundary currents, the Antarctic Circumpolar Current and equatorial zonal jets) are excluded from the comparison, since non-tidal processes (e.g., submesoscale instabilities^40,41^) are expected to play an important role in dissipation there (see Methods).

**Fig. 6:**
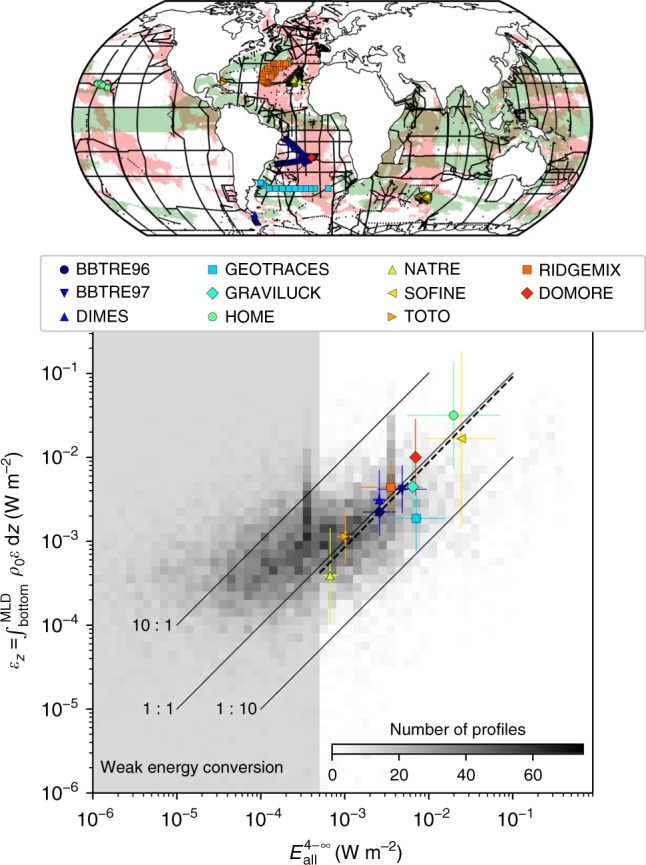
Two-dimensional histogram and scatter plot of observed energy dissipation vs. estimate of tidal energy dissipation. The *x*-axis is the theoretical estimate of energy dissipation ($E_{{\mathrm{all}}}^{4 - \infty }$) and the *y*-axis is the observed energy dissipation (*ε*_*z*_). The two-dimensional histogram (colours) is based on the rate of energy dissipation computed from a finescale parameterisation^9^ applied to vertical profiles of density and velocity located along the black lines shown in the map. The scatter plot is based on the rate of energy dissipation computed from microstructure profiles^8^ at stations shown in the top map. Data points for which energy conversion is less than 5 × 10^−4^ W m^−2^ were excluded in microstructure data and are shadowed in grey. Regions where mean kinetic energy or mesoscale eddy kinetic energy is higher than 200 cm^2^ s^−2^ are shown in green in the top map, and were excluded from the histogram and scatter plot. Regions where energy conversion exceeds 5 × 10^−4^ W m^−2^ are shown in red in the top map. The dashed line is a linear regression on logged microstructure data

The two-dimensional histogram of finescale dissipation vs. predicted near-field dissipation (Fig. 6) shows that there is a strong relationship between the two variables that approaches 1:1 wherever the conversion is significant (>5 × 10^−4^ W m^−2^, typical lower bound in regions where tidal energy conversion occurs^24^). Indeed, *r*^2^ = 0.96 for the linear regression on data binned in 0.1 log intervals. For smaller rates of conversion (shadowed area), observed dissipation exceeds the theoretical estimate, which suggests that turbulence in those areas is predominantly fuelled by other local (e.g., wind-driven) or non-local (e.g., far-field dissipation of low-mode internal tides^11^) processes. Note that in regions of rough topography, the finescale parameterisation may lose accuracy^42^ and so could be unsuitable to examine near-field dissipation.

Microstructure profilers measure microscale turbulence, and thus provide the most reliable estimates of the rate of turbulent dissipation. We consider a subset of the microstructure-derived dissipation estimates gathered by Waterhouse et al.^8^ relevant to tidally induced mixing, and augmented with the more recent RidgeMix^22^ and DoMORE^43^ data sets collected over the northern and southern Mid-Atlantic Ridge, respectively. Only stations where the predicted tidal conversion exceeds 5 × 10^−4^ W m^−2^ are examined (Fig. 6). Pictograms and associated error bars indicate the mean and standard deviation values for each cruise data set. Error bars thus encode the spatial (horizontal and vertical error bars) and temporal (vertical error bars) variability of turbulent dissipation for each expedition, where some of the variability likely stems from spring-neap cycle biases in sampling^22^ and other low-frequency, non-tidal processes. This scatter plot highlights the close connection between tidal energy conversion into high modes and local energy dissipation, as the linear regression performed on logged data gives a proportionality coefficient of 1.02 and *r*^2^ = 0.83 (dashed line in Fig. 6). Microstructure data thus brings a quantitative support to our definition of the critical mode number.

The strength of our formulation of near-field dissipation is its universality, despite the existence of seafloor topographies of very different natures across the world’s oceans. For instance, it predicts equally well the near-field dissipation over the rough, small-scale topography of the Mid-Atlantic Ridge (BBTRE96, BBTRE97, RidgeMix and DoMORE) and over the large-scale, steep ridge of Hawaii (HOME). Indeed, segregating internal tides by modes allows to take into account those differences quantitatively.

## Discussion

Our results unveil a widespread, intense generation of high-mode internal tides tied up to strong near-field dissipation. We now compare our estimate of near-field dissipation, $E_{{\mathrm{all}}}^{4 - \infty }$, to two parameterisations of near-field dissipation currently used in climate-scale ocean models. Such parameterisations are constructed from maps of barotropic-to-baroclinic tidal energy conversion for the eight principal tidal constituents. A fraction of the energy conversion, usually taken to be uniform and equal to 1/3, is assumed to fuel dissipation within the local water column. This dissipation is then distributed vertically, following an exponential decay from the seafloor upward. In the following, we ignore the vertical distribution within the water column, and focus on the maps of energy conversion providing local dissipation. The NEMO model^15^ uses 1/3 of the global estimate of energy conversion by Nycander^24^, and is hereafter denoted *E*_NEMO_. The CCSM4 model^14^ uses the parameterisation of energy conversion of Jayne and St. Laurent^44^, re-scaled as in Jayne^2^ to produce 1 TW of dissipation below 1000 m, and then multiplied by 1/3. It is hereafter denoted *E*_CCSM_.

The three estimates of local dissipation, $E_{{\mathrm{all}}}^{4 - \infty }$, *E*_NEMO_ and *E*_CCSM_, produce 606, 413, and 482 GW of energy dissipation at seafloor depths exceeding 500 m, respectively. At seafloor depths shallower than 2500 m, $E_{{\mathrm{all}}}^{4 - \infty }$ and *E*_CCSM_ are very similar (391 vs. 381 GW), but *E*_NEMO_ is weaker (261 GW). Differences become more pronounced at seafloor depths deeper than 2500 m, where $E_{{\mathrm{all}}}^{4 - \infty }$ represents 215 GW of dissipation, which is 41% larger than *E*_NEMO_ (152 GW) and 113% larger than *E*_CCSM_ (101 GW). The larger dissipation below 2500 m is consistent with large *q* values found over abyssal topography (Fig. 5).

**Fig. 7:**
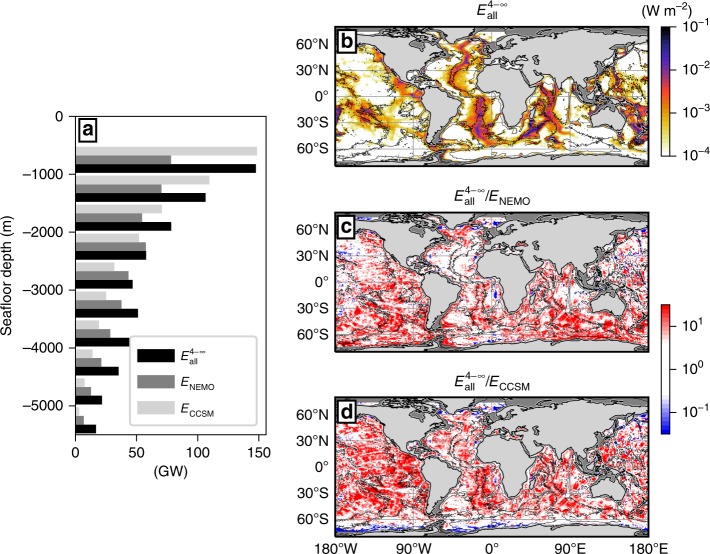
Comparison of proposed and currently used estimates of near-field tidal mixing. **a** Distribution of energy dissipation as a function of seafloor depth for the three estimates: (black) our proposed estimate, $E_{{\mathrm{all}}}^{4 - \infty }$; (dark grey) *E*_NEMO_, based on Nycander’s estimate of energy conversion and used in the NEMO model; and (light grey) *E*_CCSM_, based on Jayne and St Laurent’s estimate and used in the CCSM4 model. Maps of **b** $E_{{\mathrm{all}}}^{4 - \infty }$, and the ratio our estimate to the currently used ones, **c** $E_{{\mathrm{all}}}^{4 - \infty }/E_{{\mathrm{NEMO}}}$ and **d** $E_{{\mathrm{all}}}^{4 - \infty }/E_{{\mathrm{CCSM}}}$

The map of $E_{{\mathrm{all}}}^{4 - \infty }$ illustrates the near-field dissipation hotspots, such as mid-ocean ridges featuring rough topography (Fig. 7b). The ratio of our estimate to the others highlights two important differences (Fig. 7c, d). In regions of rough topography, our estimate gives higher levels of dissipation because of the high-mode internal tide generation that is absent from *E*_NEMO_ and *E*_CCSM_. In contrast, over steep ridges and seamounts, mostly in the Pacific basin, dissipation is comparatively weaker than for *E*_NEMO_ and *E*_CCSM_ because modes 1–3 are excluded from our estimate. We do not expect strong dissipation—relative to conversion—at the latter generation hot spots, but rather a redistribution by low modes contributing to far-field dissipation.

The patterns and magnitudes of near-field dissipation estimated in the present work thus differ substantially from those implied by current parameterisations. Implications are manifold. First, our mode-partitioned internal tide generation estimate may serve to improve the representation of wave drag, i.e., the energy extracted locally from the barotropic tide, in barotropic tide models^45^ and in climate-scale ocean models that include tidal forcing^46^. Notably, our estimate takes into account the local properties of seafloor topography, and should thereby reduce known geographical biases in barotropic tide models^45^. Second, our map of $E_{{\mathrm{all}}}^{4 - \infty }$ can provide the power input to the parameterisation of near-field internal tide-driven mixing. Its use in place of preceding maps is expected to improve the representation of deep-ocean mixing in ocean models, potentially improving the simulated overturning circulation by reconciling strong abyssal transports with slow pycnocline mixing^47^ (Fig. 7a). Overall, our revised estimate of internal tide-driven dissipation will help narrow down unknowns in the rates and energy pathways of deep-ocean mixing, and represents a significant step toward the closure of oceanic energy and diapycnal mixing budgets in observations and models.

## Methods

### Semi-analytical model of tidal energy conversion

The barotropic-to-baroclinic tide energy conversion model was formulated by Bell^19,20^, and is based on two main assumptions that enable derivation of a linear theory. First, the topographic slope, |∇*h*|, is assumed to be smaller than the slope of a radiated internal wave beam, $[(\omega ^2 - f^2)/(N_b^2 - \omega ^2)]^{1/2}$, where *ω* is the tidal frequency, *f* is the Coriolis frequency and *N*_*b*_ is the buoyancy frequency near the seafloor. The ratio of the topographic slope to the slope of a radiated beam defines the steepness parameter:

1 $$\gamma = \frac{{|\nabla h|}}{{[(\omega ^2 - f^2)/(N_b^2 - \omega ^2)]^{1/2}}}.$$

When *γ* < 1 (*γ* > 1), the topography is referred to as subcritical (supercritical). Second, the tidal excursion, *u*_0_/*ω*, where *u*_0_ is the barotropic tide velocity, is assumed to be small compared to the topographic scale 1/*k*, where *k* is a characteristic wavenumber of the underlying topography. In the deep ocean, i.e., excluding continental shelves, the major topographic structures generating internal tides are mid-ocean ridges. The assumptions of subcritical topography and small tidal excursion are valid on most of the areas covered by these structures, due to weak barotropic tidal currents [*u*_0_ = *O*(1) cm s^−1^] and weak stratification that allows beams to propagate in a direction close to the vertical.

We used St. Laurent and Garrett’s^21^ formulation of tidal energy conversion, *E*_*ω*_, at a fundamental tidal frequency *ω*:

2 $$\begin{array}{c}E_\omega (K,\theta ) = \frac{1}{2}\rho _0\frac{{\left[ {(N_b^2 - \omega ^2)(\omega ^2 - f^2)} \right]^{1/2}}}{\omega }\\ \times \left( {u_e^2\cos ^2\theta + v_e^2\sin ^2\theta } \right)K\phi (K,\theta ).\end{array}$$

In this equation, *N*_*b*_ is the buoyancy frequency close to the bottom computed from the World Ocean Atlas 2013 (WOA13^48,49^); *u*_*e*_ (*v*_*e*_) is the barotropic tidal velocity amplitude from TPXO8^50^, in the direction of the semimajor (semiminor) axis of the tidal ellipse [(*x*_*e*_, *y*_*e*_) coordinate system]; $K = (k_x^2 + k_y^2)^{1/2}$ is the total horizontal wavenumber, with *k*_*x*_ and *k*_*y*_ being the horizontal wavenumbers in the (*x*_*e*_, *y*_*e*_) coordinate system; and *θ* = arctan(*k*_*y*_/*k*_*x*_). The two-dimensional power spectrum of topography, *ϕ*, is computed from the Shuttle Radar Topography Mission dataset (SRTM30_PLUS^51^). SRTM30_PLUS is a global bathymetry dataset at a 30-s resolution based on the 1-min Smith and Sandwell^52^ bathymetry, and incorporates higher-resolution data from ship soundings wherever available. *ϕ* is normalised to satisfy ${\int}_0^{2\pi } {{\int}_0^\infty \phi } (K,\theta )K \, \:{\mathrm{d}}K \, \:{\mathrm{d}}\theta = \overline {h^2}$, where $\overline {h^2}$ is the mean square height of topography.

The equivalent wavenumber of mode *j* is

3 $$K_j = \frac{{j\pi (\omega ^2 - f^2)^{1/2}}}{{N_0b}}.$$

*N*_0_ and *b* are parameters of an exponential fit to the buoyancy frequency *N* = *N*_0_ exp(*z*/*b*). This enables computation of the energy flux into mode *j* as

4 $$E_\omega ^j = {\int}_0^{2\pi } {{\int}_{K_j - \delta K/2}^{K_j + \delta K/2} {E_\omega } } (K,\theta )K \, \:{\mathrm{d}}K \, \:{\mathrm{d}}\theta \;({\mathrm{W}}\:{\mathrm{m}}^{ - {\mathrm{2}}}),$$

where *δK* = *K*_2_ − *K*_1_. The total energy flux is then

5 $$E_\omega ^t = {\int}_0^{2\pi } {{\int}_{K_1}^\infty {E_f} } (K,\theta )K \, \:{\mathrm{d}}K \, \:{\mathrm{d}}\theta \;({\mathrm{W}}\:{\mathrm{m}}^{ - {\mathrm{2}}}),$$

where the lower boundary of integration in wavenumber space is the mode-1 equivalent wavenumber, *K*_1_, to take into account the finite depth of the ocean.

We computed *E*_*ω*_ for *ω* ∈ {M_2_, S_2_, K_1_} on a global grid of 1/2° resolution. A supercritical-slope correction was made a posteriori (see Supplementary Note 1). We only considered the points where the bathymetry is deeper than 500 m. At shallower ocean depths, topographic slopes are more likely to be supercritical due to enhanced stratification, and tidal currents are stronger due to mass continuity, hence potentially violating the small tidal excursion assumption.

### Barotropic tide energy dissipation

The dissipation rate of the barotropic tide, *D*_*ω*_, at the tidal frequency *ω* can be computed as^53^

6 $$D_\omega = W - \nabla \cdot {\mathbf{P}}\;[{\mathrm{W}}\:{\mathrm{m}}^{ - {\mathrm{2}}}],$$

where *W* is the work done by the barotropic tide, and **P** is the barotropic tide energy flux. **P** is defined as

7 $${\mathbf{P}} = \rho _0g\langle {\mathbf{U}}\zeta \rangle ,$$

where *ζ* is the tidal elevation, and **U** is the barotropic tide volume transport, both extracted from TPXO8. *W* is defined as

8 $$W = \rho _0g\left\langle {{\mathbf{U}}\cdot \nabla (\zeta _{{\mathrm{eq}}} + \zeta _{{\mathrm{sal}}})} \right\rangle ,$$

where *ζ*_eq_ is the equilibrium tidal elevation, and *ζ*_sal_ is the tidal elevation induced by the tide’s self-attraction and loading^54^.

### Mode-1 M_2_ energy fluxes from satellite altimetry

Mode-1 M_2_ internal-tide horizontal energy flux at a horizontal resolution of 1/10° from Zhao et al.^27^ was used in this study to quantify the generation of M_2_ internal tides. A two-dimensional plane wave fit method is applied to extract internal tides from satellite SSH, and perform a modal decomposition that enables inference of mode-1 internal tide pressure from SSH. Assuming that the energy partition between potential and kinetic energy components depends only on latitude and tidal frequency, the internal tide velocity is also estimated from SSH. Finally, the vertically integrated horizontal energy flux, ${\mathbf{F}}_{{\mathrm{M}}_2}^1$, is computed. Positive divergence of the horizontal energy flux, denoted as $(\nabla \cdot {\mathbf{F}}_{{\mathrm{M}}_2}^1)^ +$ in the article, indicates regions of mode-1 internal tide generation^22^.

### Global HYCOM simulation

HYCOM (Hybrid Coordinate Ocean Model) is the operational ocean forecast model used by the United States Navy^55^. The simulation considered in this study was run with realistic atmospheric forcing from the NAVy Global Environmental Model (NAVGEM^56^) and astronomical tidal forcing. The model was run in a forward (non-data-assimilative) mode at 1/25 degree (4 km) nominal horizontal resolution, with 41 layers in the vertical, using a hybrid vertical coordinate that is isopycnal in the open ocean, uses z-layers in the mixed layer, and transitions to terrain-following in shallow water. Hourly 3-d fields were saved from September 2016; 15 days of this period were analysed for this paper. Data were interpolated to 25-m depth intervals in the vertical and harmonic fits were applied to extract the M_2_ component of the baroclinic velocities and potential density at each depth level. Vertical normal modes were computed from the time-averaged stratification profile by solving the Sturm-Liouville problem. The 4 km model resolution effectively limits the resolved modes to the first 5 baroclinic modes. Modal barotropic-to-baroclinic conversion values were computed from the modal perturbation pressure amplitudes, bathymetry, and barotropic velocity^57^.

### Theoretical estimate of near-field energy dissipation

We computed the internal tide generation for the M_2_, S_2_ and K_1_ tides, which together account for 90% of the total energy conversion summed over the eight principal constituents^24^ (M_2_, S_2_, N_2_, K_2_, K_1_, O_1_, P_1_, Q_1_). Patterns of internal tide generation barely change for tides at close frequencies^26^. We therefore used $E_{{\mathrm{M}}_2}$ and $E_{{\mathrm{S}}_2}$ as proxies for $E_{{\mathrm{N}}_2}$ and $E_{{\mathrm{K}}_2}$, respectively, and $E_{{\mathrm{K}}_1}$ as a proxy for $E_{{\mathrm{O}}_{1}}$, $E_{{\mathrm{P}}_1}$ and $E_{{\mathrm{Q}}_1}$. We then applied a scaling factor set by the power ratios^58^ to obtain the near-field energy dissipation of the principal eight components:

9 $$E_{{\mathrm{all}}}^{4 - \infty } = 1.05 \times E_{{\mathrm{M}}_2}^{4 - \infty } + 1.09 \times E_{{\mathrm{S}}_{2}}^{4 - \infty } + 1.70 \times E_{{\mathrm{K}}_{1}}^{4 - \infty }$$

### Finescale parameterisation of energy dissipation

We used the finescale parameterisation of the rate of energy dissipation, *ε* (W kg^−1^), computed by Kunze^9^, and available at ftp://ftp.nwra.com/outgoing/kunze/iwturb. The parameterisation is based on vertical strain applied to 27,218 hydrographic profiles. We discarded profiles covering <80% of the water column, which corresponds to 35% of the database. *ε* was then integrated vertically and multiplied by *ρ*_0_ = 1025 kg m^−3^ to give *ε*_*z*_ (W m^−2^), used in Fig. 6. Only 0.4% of the profiles cover depths shallower than 100 m so we can reasonably assume that mixed-layer processes do not interfere in the dissipation signal.

### Microstructure estimates of energy dissipation

We used the microstructure estimates of energy dissipation *ε* (W kg^−1^) from different cruises gathered by Waterhouse et al.^8^ and available at https://microstructure.ucsd.edu. We only considered data relevant to tidally induced mixing, i.e., we removed data collected in regions of insignificant internal tide generation, and we added data from the RidgeMix^22^ and DoMORE^43^ cruises. We discarded profiles covering <60% of the water column (32% of the whole dataset), which leaves us with 476 profiles. *ε* was vertically integrated from the deepest point to the base of the mixed layer, defined by a drop of temperature of 0.2 °C from the temperature at 10 m depth, following de Boyer Montégut et al.^59^ Finally, we multiplied each vertically integrated value by *ρ*_0_ = 1025 kg m^−3^ to give *ε*_*z*_ (W m^−2^) used in Fig. 6.

### Mean and eddy kinetic energy from satellite altimetry

Mean and eddy kinetic energy (MKE and EKE) were computed from surface geostrophic velocity derived from the Absolute Dynamic Topography (ADT) measured by satellite altimetry. Velocity fields were downloaded from https://www.aviso.altimetry.fr/en/home.html. ${\mathrm{MKE}} = \frac{1}{2}(\bar u^2 + \bar v^2)$ was computed from the mean velocity ($\bar u,\bar v$) over the period 2000–2014, and ${\mathrm{EKE}} = \frac{1}{2}(\overline {{u\prime}^2} + \overline {{v\prime}^2} )$ was computed from the eddy velocity ($u{\prime},v{\prime} = u - \bar u,v - \bar v$).

## Supplementary information

Supplementary Information

## Data Availability

The tidal energy conversion from the semi-analytical model is available upon request.

## Code availability

The code for the barotropic-to-baroclinic tidal energy conversion model is available upon request.

## References

[1] Polzin K, Toole J, Ledwell J, Schmitt R (1997). Spatial variability of turbulent mixing in the abyssal ocean. Science.

[2] Jayne SR (2009). The impact of abyssal mixing parameterizations in an ocean general circulation model. J. Phys. Oceanogr..

[3] de Lavergne C, Madec G, Le Sommer J, Nurser AG, Naveira Garabato AC (2016). On the consumption of Antarctic Bottom Water in the abyssal ocean. J. Phys. Oceanogr..

[4] Melet A, Legg S, Hallberg R (2016). Climatic impacts of parameterized local and remote tidal mixing. J. Clim..

[5] Munk W, Wunsch C (1998). Abyssal recipes II: energetics of tidal and wind mixing. Deep-Sea Res. I.

[6] Ferrari R, Wunsch C (2009). Ocean circulation kinetic energy: reservoirs, sources, and sinks. Annu. Rev. Fluid Mech..

[7] MacKinnon JA (2017). Climate process team on internal-wave driven ocean mixing. Bull. Am. Meteor. Soc..

[8] Waterhouse AF (2014). Global patterns of diapycnal mixing from measurements of the turbulent dissipation rate. J. Phys. Oceanogr..

[9] Kunze E (2017). Internal-wave-driven mixing: Global geography and budgets. J. Phys. Oceanogr..

[10] Garrett C, Kunze E (2007). Internal tide generation in the deep ocean. Annu. Rev. Fluid Mech..

[11] Alford MH (2003). Redistribution of energy available for ocean mixing by long-range propagation of internal waves. Nature.

[12] Nikurashin M, Legg S (2011). A mechanism for local dissipation of internal tides generated at rough topography. J. Phys. Oceanogr..

[13] St Laurent L, Simmons H, Jayne S (2002). Estimating tidally driven mixing in the deep ocean. Geophys. Res. Lett..

[14] Danabasoglu G (2012). The CCSM4 ocean component. J. Clim..

[15] Madec, G. et al. NEMO ocean engine. Tech. Rep. (2015).

[16] St Laurent L, Nash J (2004). An examination of the radiative and dissipative properties of deep ocean internal tides. Deep-Sea Res. II.

[17] Falahat S, Nycander J, Roquet F, Thurnherr AM, Hibiya T (2014). Comparison of calculated energy flux of internal tides with microstructure measurements. Tellus A.

[18] Lefauve A, Muller C, Melet A (2015). A three-dimensional map of tidal dissipation over abyssal hills. J. Geophys. Res. Oceans.

[19] Bell T (1975). Lee waves in stratified flows with simple harmonic time dependence. J. Fluid Mech..

[20] Bell T (1975). Topographically generated internal waves in the open ocean. J. Geophys. Res..

[21] St. Laurent L, Garrett C (2002). The role of internal tides in mixing the deep ocean. J. Phys. Oceanogr..

[22] Vic C (2018). The lifecycle of semidiurnal internal tides over the northern Mid-Atlantic Ridge. J. Phys. Oceanogr..

[23] Falahat S, Nycander J, Roquet F, Zarroug M (2014). Global calculation of tidal energy conversion into vertical normal modes. J. Phys. Oceanogr..

[24] Nycander, J. Generation of internal waves in the deep ocean by tides. *J. Geophys. Res*. **110**, C10028 (2005).

[25] Melet A (2013). Internal tide generation by abyssal hills using analytical theory. J. Geophys. Res. Oceans.

[26] Egbert, G. D. & Ray, R. D. Semi-diurnal and diurnal tidal dissipation from TOPEX/Poseidon altimetry. *Geophys. Res. Lett*. **30**, 1907 (2003).

[27] Zhao Z, Alford MH, Girton JB, Rainville L, Simmons HL (2016). Global observations of open-ocean mode-1 M_2_ internal tides. J. Phys. Oceanogr..

[28] Ray RD, Cartwright DE (2001). Estimates of internal tide energy fluxes from Topex/Poseidon altimetry: Central North Pacific. Geophys. Res. Lett..

[29] Buijsman MC (2016). Impact of parameterized internal wave drag on the semidiurnal energy balance in a global ocean circulation model. J. Phys. Oceanogr..

[30] Rudnick DL (2003). From tides to mixing along the Hawaiian Ridge. Science.

[31] Merrifield MA, Holloway PE (2002). Model estimates of M_2_ internal tide energetics at the Hawaiian Ridge. J. Geophys. Res..

[32] Rainville L, Pinkel R (2006). Propagation of low-mode internal waves through the ocean. J. Phys. Oceanogr..

[33] Alford MH, Zhao Z (2007). Global patterns of low-mode internal-wave propagation. Part I: Energy and energy flux. J. Phys. Oceanogr..

[34] Zhao Z, Alford MH, MacKinnon JA, Pinkel R (2010). Long-range propagation of the semidiurnal internal tide from the Hawaiian Ridge. J. Phys. Oceanogr..

[35] Olbers DJ (1983). Models of the oceanic internal wave field. Rev. Geophys..

[36] Ansong JK (2017). Semidiurnal internal tide energy fluxes and their variability in a Global Ocean Model and moored observations. J. Geophys. Res. Oceans.

[37] Legg S, Klymak J (2008). Internal hydraulic jumps and overturning generated by tidal flow over a tall steep ridge. J. Phys. Oceanogr..

[38] Klymak JM, Pinkel R, Rainville L (2008). Direct breaking of the internal tide near topography: Kaena Ridge, Hawaii. J. Phys. Oceanogr..

[39] Van Haren, H. & Gostiaux, L. A deep-ocean Kelvin-Helmholtz billow train. *Geophys. Res. Lett*. **37**, L03605 (2010).

[40] D’Asaro E, Lee C, Rainville L, Thomas L, Harcourt R (2011). Enhanced turbulence and energy dissipation at ocean fronts. Science.

[41] Gula J, Molemaker MJ, McWilliams JC (2016). Topographic generation of submesoscale centrifugal instability and energy dissipation. Nat. Commun..

[42] Liang CR, Shang XD, Qi YF, Chen GY, Yu LH (2018). Assessment of fine-scale parameterizations at low latitudes of the North Pacific. Sci. Rep..

[43] Clément L, Thurnherr AM, St. Laurent LC (2017). Turbulent mixing in a deep fracture zone on the Mid-Atlantic Ridge. J. Phys. Oceanogr..

[44] Jayne SR, St Laurent LC (2001). Parameterizing tidal dissipation over rough topography. Geophys. Res. Lett..

[45] Egbert, G. D., Ray, R. D. & Bills, B. G. Numerical modeling of the global semidiurnal tide in the present day and in the last glacial maximum. *J. Geophys. Res. Oceans* **109**, C03003 (2004).

[46] Arbic BK, Garner ST, Hallberg RW, Simmons HL (2004). The accuracy of surface elevations in forward global barotropic and baroclinic tide models. Deep-Sea Res. II.

[47] de Lavergne C, Madec G, Roquet F, Holmes R, McDougall T (2017). Abyssal ocean overturning shaped by seafloor distribution. Nature.

[48] Locarnini R (2013). World Ocean Atlas 2013, Volume 1: Temperature. NOAA Atlas NESDIS.

[49] Zweng M (2013). World Ocean Atlas 2013, Volume 2: Salinity. NOAA Atlas NESDIS.

[50] Egbert GD, Erofeeva SY (2002). Efficient inverse modeling of barotropic ocean tides. J. Atmos. Ocean. Technol..

[51] Becker J (2009). Global bathymetry and elevation data at 30 arc seconds resolution: SRTM30_PLUS. Mar. Geod..

[52] Smith W, Sandwell D (1997). Global sea floor topography from satellite altimetry and ship depth soundings. Science.

[53] Egbert G, Ray R (2000). Significant dissipation of tidal energy in the deep ocean inferred from satellite altimeter data. Nature.

[54] Ray R (1998). Ocean self-attraction and loading in numerical tidal models. Mar. Geod..

[55] Metzger EJ (2014). US Navy operational global ocean and Arctic ice prediction systems. Oceanography.

[56] Hogan TF (2014). The navy global environmental model. Oceanography.

[57] Buijsman MC (2014). Three-dimensional double-ridge internal tide resonance in Luzon Strait. J. Phys. Oceanogr..

[58] de Lavergne C (2019). Toward global maps of internal tide energy sinks. Ocean Model..

[59] de Boyer Montégut, C., Madec, G., Fischer, A. S., Lazar, A. & Iudicone, D. Mixed layer depth over the global ocean: An examination of profile data and a profile-based climatology. *J. Geophys. Res. Oceans* **109**, C12003 (2004).

[60] Falahat, S. et al. Global estimates of internal tide generation rates at 1/30° resolution. *SEANOE.* 10.58153/58153 (2018).

