# Supplementary material for: Deep-ocean mixing driven by small-scale internal tides
Source: Nat Commun. 2019 May 8;10:2099. doi: 10.1038/s41467-019-10149-5 (PMC6506475; doi:10.1038/s41467-019-10149-5)
Supplement: Supplementary file 1 — Supplementary Information [file 41467_2019_10149_MOESM1_ESM.pdf]

Deep-ocean mixing driven by small-scale internal tides – Supplementary note

Clément Vic[1], Alberto C. Naveira Garabato[1], J. A. Mattias Green[2], Amy F. Waterhouse[3], Zhongxiang Zhao[4], Angélique Melet[5], Casimir de Lavergne[6], Maarten C. Buijsman[7] & Gordon R. Stephenson[7]

[1] *Ocean and Earth Science, University of Southampton, National Oceanography Centre, Southampton, United Kingdom*

[2] *School of Ocean Sciences, Bangor University, Menai Bridge, Anglesey, United Kingdom*

[3] *Scripps Institution of Oceanography, University of California San Diego, La Jolla, California*

[4] *Applied Physics Laboratory, University of Washington, Seattle, Washington*

[5] *Mercator Ocean, Ramonville Saint-Agne, France*

[6] *LOCEAN Laboratory, Sorbonne Université-CNRS-IRD-MNHN, Paris, France*

[7] *University of Southern Mississippi, Stennis Space Center, Mississippi*

Supplementary Note 1

Supercritical-slope correction for barotropic-to-baroclinic energy conversion.

Although supercritical slopes cover a small fraction of the total seafloor area ($\sim$1%), they accumulate between 500 m and 1500 m (reaching up to 20% of the global seafloor area at a given depth, Supplementary Figure 1a). This is where tidal energy conversion is the strongest (Supplementary Figure 1b), noticeably due to enhanced stratification in the thermocline and steep topographic slopes of continental shelf breaks and isolated seamounts. As such, the modelled energy conversion, which is not formally valid for $\gamma > 1$, must be corrected in these areas. We opted for a correction à la Melet et al.[1] and Falahat et al.[2] – inspired by Nycander[3] –, where E_ω is divided by γ^2 wherever $\gamma > 1$. The correction is at work at depths shallower than 2000 m (Figs. 1b,c) and overall reduces the total conversion rate below 500 m from 912 GW to 787 GW.

Despite the correction, the calculation gives a very few unrealistic values at shallow depths, mostly in the western Pacific. In these areas, uncertainties in the calculation arise from a lack of hydrographic data, and less reliable tidal velocities, as noticed in [4]. To circumvent this caveat, we use a capping at 1 W m^{-2}, as commonly done in previous studies[1,2,5]. This capping only affects regions shallower than 700 m (Figs. 1b,c), and further reduces the global conversion rate below 500 m to 737 GW. In the article, energy conversion is systematically corrected and capped, and budgets exclude depths shallower than 700 m to discard most of the calculation suffering from supercritical-slope correction and capping.

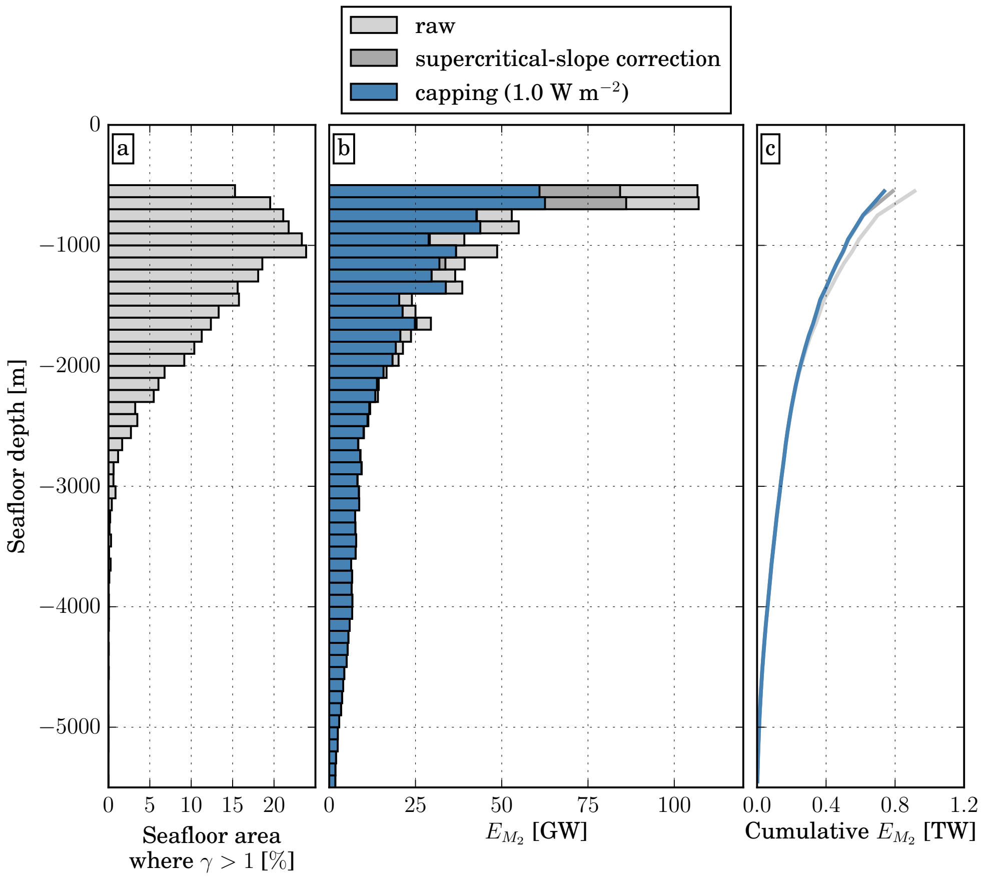

Supplementary Figure 1: (a) Seafloor area covered by supercritical slopes at a given depth, (b) energy conversion E_{M_2} binned as a function of seafloor depth and (c) cumulated from the deepest bin, for the (light gray) raw calculation, and calculation corrected (dark gray) for supercritical slopes and (blue) for supercritical slopes and capped at 1 $\mathrm{W\,m^{-2}}$.

Supplementary Note 2

Comparison of D_{M_2} and $E_{M_2}^{1-\infty}$.

Supplementary Figure 2 shows that D_{M_2} and $E_{M_2}^{1-\infty}$ are well correlated (regression coefficient is

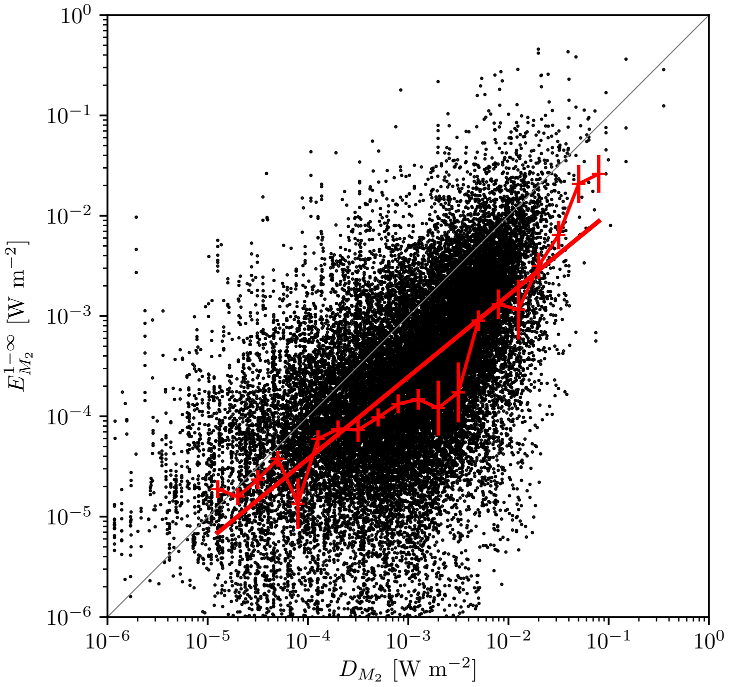

Supplementary Figure 2: Scatter plot of the barotropic tide dissipation D_{M_2} and total energy conversion $E_{M_2}^{1-\infty}$. Thin red line is the binned data on 0.2 log intervals and error bars are the associated standard deviations. Linear regression on the binned data is shown by thick red line. Gray line shows the 1:1 ratio.

$r^2 = 0.90$), i.e., hotspots of barotropic tide dissipation are co-located with hotspots of barotropic-to-baroclinic tide energy conversion. We also computed the coefficient of determination r^2 relative

to the $y = x$ fit such as:

$$r^2 = 1 - \frac{\sum_i (y_i - f_i)^2}{\sum_i (y_i - \bar{y})^2}, \tag{1}$$

where y_i is the observed field (D_{M_2}), $\bar{y}$ is its mean, and f_i is the 'prediction' ($E_{M_2}^{1-\infty}$). We found $r^2_{y=x} = 0.48$, which means that $E_{M_2}^{1-\infty}$ is a good prediction for D_{M_2}.

Supplementary Note 3

Comparison of $(\nabla \cdot \mathbf{F}^1_{M_2})^+$ and $E^1_{M_2}$.

Supplementary Figure 3 shows that $(\nabla \cdot \mathbf{F}^1_{M_2})^+$ and $E^1_{M_2}$ are well correlated (regression coefficient

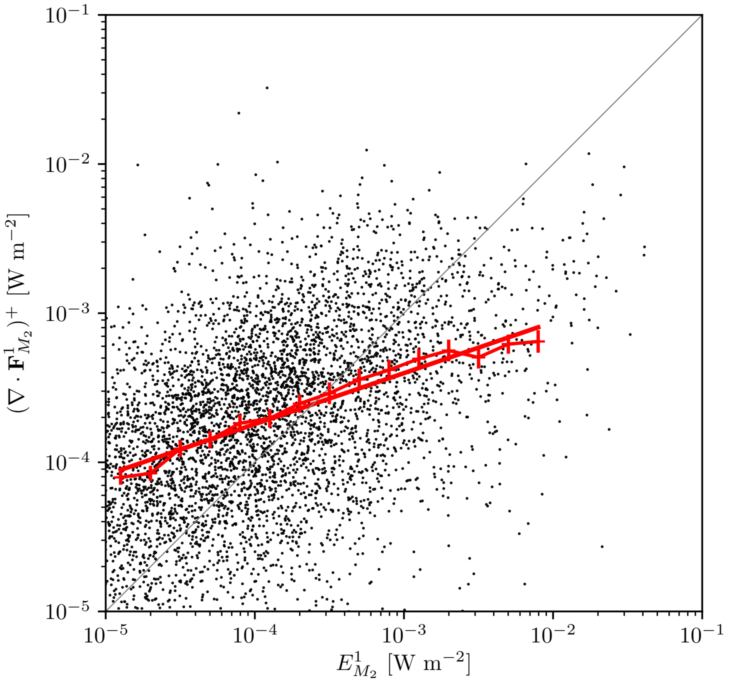

Supplementary Figure 3: Scatter plot of the divergence of mode-1 energy flux from satellite altimetry $(\nabla \cdot \mathbf{F}^1_{M_2})^+$ (Zhao et al.[6]) and energy conversion into mode 1 $E^1_{M_2}$. Thin red line is the binned data on 0.2 log intervals and error bars are the associated standard deviations. Linear regression on the binned data is shown by thick red line.

is $r^2 = 0.94$), i.e., observed and predicted hotspots of mode-1 internal tide generation are co-located. However, we found a negative $r^2_{y=x}$, due to the relatively strong deviation of the data from $y = x$ line. In fact, $E^1_{M_2}$ seems to overestimate (underestimate) $(\nabla \cdot \mathbf{F}^1_{M_2})^+$ for weak (strong) generation, i.e., red line above (below) $y = x$ (Supplementary Figure 3).

Supplementary Note 4

Sensitivity of the critical mode number and q on the attenuation length scale.

The critical mode number n_{crit} (and q, subsequently) depends on the attenuation length scale of mode 1, L_1, which is expected to vary geographically. However, L_1 is impossible to estimate globally since the only in situ observations sample a beam emanating from the Hawaiian Ridge. To test the sensitivity of n_{crit} on L_1, we doubled or halved our conservative estimate of L_1 (1300 km) and find that the critical mode number only varies by one unit (Supplementary Figure 4). This is due to the sharp decay of L_n with mode number ($L_n \approx L_1/n^3$). Specifically, we find that $n_{crit} = 3, 4, 5$ for $L_1 = 650, 1300, 2600$ km, respectively. It is thus reasonable to assume that n_{crit} varies globally within a small range of values, say {3,4,5}.

Furthermore, the value of q is not very sensitive on n_{crit} varying in {3,4,5}. This partly stems from our result that modes > 10 represent an important part of the barotropic-to-baroclinic tide conversion. Consequently, the individual contributions of modes 3,4,5 are little compared to the total. To illustrate this point, we picked two data points in our model where strong internal tides of different character are generated : one over the Hawaiian Ridge (23.0°N, 162.0°W) and one over the Mid-Atlantic Ridge (MAR) in the Brazil Basin (33.5°S, 15.0°W). Supplementary Figure 5-left shows the cumulative energy flux as a function of mode number for these two locations. In Hawaii, mode 1 strongly dominates the flux (46%) whereas in the MAR, modes > 10 altogether dominate (40%); yet, in both regions, the individual contributions of modes 3,4,5 are little. Hence, q is weakly sensitive on $n_{crit} \in \{3, 4, 5\}$ (Supplementary Figure 5-right). q is in the range 0.59-

0.74 over the MAR and 0.21-0.27 over the Hawaiian Ridge, quantitatively reflecting the systemic difference between those ridges.

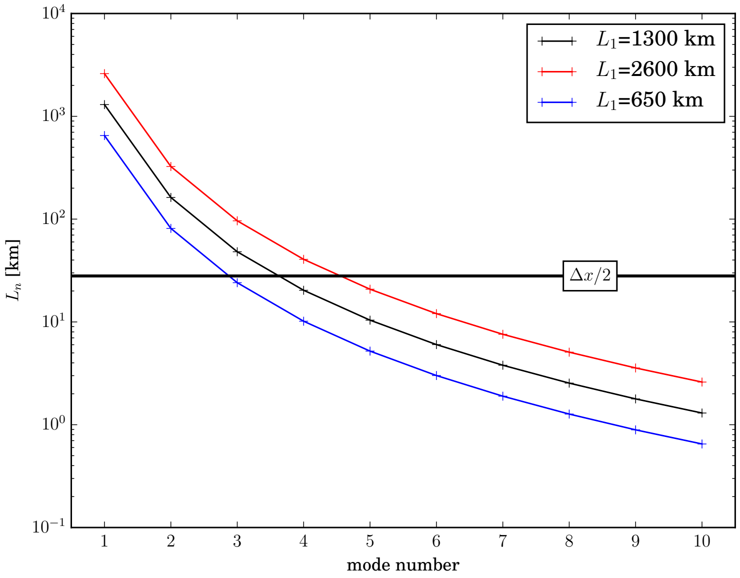

Supplementary Figure 4: Attenuation length scale L_n as a function of mode number for three different values of L_1 (see legend). The thick black line represents half the grid size.

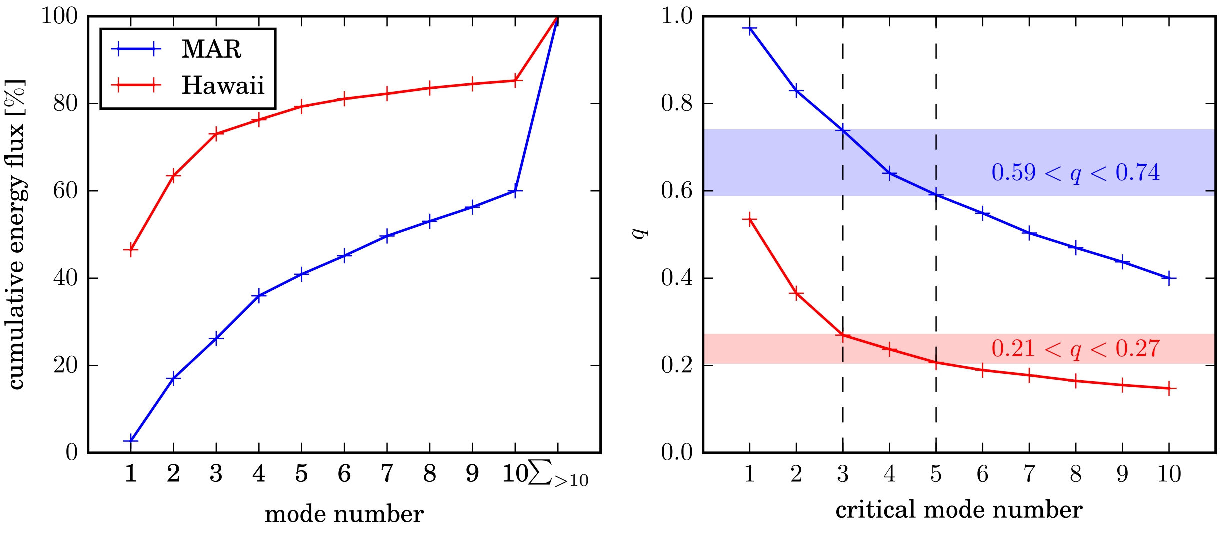

Supplementary Figure 5: (left) Cumulative energy flux as a function of mode number, in percentage of the total, and (right) q as a function of critical mode number, for pointwise conversion over the Mid-Atlantic Ridge (MAR, blue lines) and the Hawaiian Ridge (red lines).

References

1. Melet, A. *et al.* Internal tide generation by abyssal hills using analytical theory. *J. Geophys. Res. Oceans* **118**, 6303–6318 (2013).

2. Falahat, S., Nycander, J., Roquet, F. & Zarroug, M. Global calculation of tidal energy conversion into vertical normal modes. *J. Phys. Oceanogr.* **44**, 3225–3244 (2014).

3. Nycander, J. Tidal generation of internal waves from a periodic array of steep ridges. *J. Fluid Mech.* **567**, 415–432 (2006).

4. Nycander, J. Generation of internal waves in the deep ocean by tides. *J. Geophys. Res.* **110** (2005).

5. Green, J. M. & Nycander, J. A comparison of tidal conversion parameterizations for tidal models. *J. Phys. Oceanogr.* **43**, 104–119 (2013).

6. Zhao, Z., Alford, M. H., Girton, J. B., Rainville, L. & Simmons, H. L. Global observations of open-ocean mode-1 M_2 internal tides. *J. Phys. Oceanogr.* **46**, 1657–1684 (2016).
